# Study protocol: Identifying transcriptional regulatory alterations of chronic effects of blast and disturbed sleep in United States Veterans

**DOI:** 10.1371/journal.pone.0301026

**Published:** 2024-03-27

**Authors:** Molly J. Sullan, Kelly A. Stearns-Yoder, Zhaoyu Wang, Andrew J. Hoisington, Adam D. Bramoweth, Walter Carr, Yongchao Ge, Hanga Galfalvy, Fatemah Haghighi, Lisa A. Brenner

**Affiliations:** 1 VA Rocky Mountain Mental Illness Research Education and Clinical Center (MIRECC), Rocky Mountain Regional VA Medical Center (RMRVAMC), Aurora, CO, United States of America; 2 Department of Physical Medicine & Rehabilitation, University of Colorado Anschutz Medical Campus, Aurora, CO, United States of America; 3 James J. Peters VA Medical Center, Medical Epigenetics, Bronx, NY, United States of America; 4 Nash Family Department of Neuroscience, Icahn School of Medicine at Mount Sinai, New York, NY, United States of America; 5 Department of Systems Engineering & Management, Air Force Institute of Technology, Wright Patterson AFB, OH, United States of America; 6 Mental Illness Research, Education and Clinical Center (MIRECC), VA Pittsburgh Healthcare System, Pittsburgh, PA, United States of America; 7 Center for Health Equity Research and Promotion (CHERP), VA Pittsburgh Healthcare System, Pittsburgh, PA, United States of America; 8 Center for Military Psychiatry and Neuroscience, Walter Reed Army Institute of Research, Silver Spring, MD, United States of America; 9 Department of Neurology, Icahn School of Medicine at Mount Sinai, New York, NY, United States of America; 10 Departments of Psychiatry and Biostatistics, Columbia University, New York, NY, United States of America; 11 Departments of Psychiatry and Neurology, University of Colorado Anschutz Medical Campus, Aurora, CO, United States of America; University of Florida, UNITED STATES

## Abstract

Injury related to blast exposure dramatically rose during post-911 era military conflicts in Iraq and Afghanistan. Mild traumatic brain injury (mTBI) is among the most common injuries following blast, an exposure that may not result in a definitive physiologic marker (e.g., loss of consciousness). Recent research suggests that exposure to low level blasts and, more specifically repetitive blast exposure (RBE), which may be subconcussive in nature, may also impact long term physiologic and psychological outcomes, though findings have been mixed. For military personnel, blast-related injuries often occur in chaotic settings (e.g., combat), which create challenges in the immediate assessment of related-injuries, as well as acute and post-acute sequelae. As such, alternate means of identifying blast-related injuries are needed. Results from previous work suggest that epigenetic markers, such as DNA methylation, may provide a potential stable biomarker of cumulative blast exposure that can persist over time. However, more research regarding blast exposure and associations with short- and long-term sequelae is needed. Here we present the protocol for an observational study that will be completed in two phases: Phase 1 will address blast exposure among Active Duty Personnel and Phase 2 will focus on long term sequelae and biological signatures among Veterans who served in the recent conflicts and were exposed to repeated blast events as part of their military occupation. Phase 2 will be the focus of this paper. We hypothesize that Veterans will exhibit similar differentially methylated regions (DMRs) associated with changes in sleep and other psychological and physical metrics, as observed with Active Duty Personnel. Additional analyses will be conducted to compare DMRs between Phase 1 and 2 cohorts, as well as self-reported psychological and physical symptoms. This comparison between Service Members and Veterans will allow for exploration regarding the natural history of blast exposure in a quasi-longitudinal manner. Findings from this study are expected to provide additional evidence for repetitive blast-related physiologic changes associated with long-term neurobehavioral symptoms. It is expected that findings will provide foundational data for the development of effective interventions following RBE that could lead to improved long-term physical and psychological health.

## Introduction

Injuries from exposure to explosive blasts rose dramatically during post-911 military conflicts in Iraq and Afghanistan, and estimates of blast injury among Service Members in these conflicts are thought to be higher than in any previous war era [1–3]. Mild traumatic brain injury (mTBI) has been identified as one of the most common injuries following blast exposure [4], and has been the focus of much of the research in this area. In particular, exposure to low-level blasts might result in unrecognized and unreported mTBI. However, recent work has begun to identify short- and long-term sequelae resulting from repetitive blast exposure (RBE), which may be subconcussive in nature (e.g., the exposed individual does not meet diagnostic criteria for mTBI). While findings have been largely mixed, there is evidence to suggest physiologic changes (e.g., neuroinflammation [5], neurodegeneration [6], vestibular changes [7], and cognitive and behavioral sequelae [8] following low-level blast exposure in animal studies (see Belding et al., 2021 [9]). In human studies, associations have also been identified between history of repeated low level blast (LLB) exposures and changes in cognitive [10] and neuromotor functioning [11], neurobehavioral symptoms (e.g., sleep disturbance) [12], and reduced quality of life [12], though findings have been somewhat inconsistent [9].

Blast-related head injuries have also been historically difficult to assess and diagnose, as they are often marked by a lack of immediate physiologic or neurologic symptoms [13]. For military Service Members in particular, blast exposures often occur in chaotic settings (e.g., combat zones) where identification of related injuries, as well as acute sequelae are difficult to ascertain, thereby rendering the longitudinal study of symptoms challenging. Also, many Service Members experience multiple and different types of blast exposures throughout their careers, including events such as high-level blasts (e.g., being in close proximity to an improvised explosive device; IED), low-level blasts, and exposures to heavy caliber weapons. The variability in findings to date has been attributed, in part, to differences in study design, operational definitions used, and small sample sizes. Additionally, given the focus on blast-related TBI, acute and long-term effects of RBE have been largely understudied. Gaining a better understanding for how these different types of exposures interact and contribute to long-term health-related outcomes would represent an important step in determining how to adequately treat symptoms both in the acute and chronic periods. However, our understanding of the effects of different types of blast and the relationships between associated injury and long-term outcomes remains limited.

While recent work investigating outcomes following blast exposure in operational settings has begun to address this knowledge gap, studies have primarily focused on breachers, which are a unique population of military personnel who are, by definition, generally exposed to low level RBE during explosive breaching in both operational and training settings. Beyond explosive breaching, many other service members in high-risk occupations (HROs) can be repeatedly exposed to blast through multiple mechanisms, such as indirect fire (artillery, mortar), and antiarmor weapon operations [14, 15] (Table 1 [16]) in both training and combat environments [17].

**Table 1:** Representative high-risk occupations obtained from the Department of Army Pamphlet 611–21 [1,6].

High-Risk Occupation Categories	High-Risk Occupation Examples
Ammunition and Explosive Ordnance Disposal	C 89D3O-Explosive Ordnance; Specialist, CMF 89
Field Artillery	C 13B1O-Cannon Crewmember, CMF 13
Infantry	C 11B3O-Infantryman, CMF 11
Tank and Assault Amphibious Vehicle	C 91A3O-Abrams Tank Systems Maintainer

Determining which HROs across military branches impart greater risk for blast exposure is an area of continued investigation [14, 18, 19], and thus using occupational specialty as a proxy for targeted studies on blast exposure is difficult. Though within the Army [14] and Marines [15] some occupational specialties have been identified as inherently high-risk, there is limited research providing an operational definition for high-risk occupations across military branches. This type of risk classification is also complicated by factors such as the era of service (e.g., war versus peacetime), length of time within a specific occupational specialty, nature of deployments, intensity of blast exposure, level of exposure during training events, and access to protective equipment at the time of the blast. The forementioned issues all change the risk profile associated within a given occupational specialty. While work is being done to better categorize and understand occupational blast exposure in the military [14, 15, 20, 21], there remains no gold standard for classifying HROs across branches of the military.

Additionally, the mechanism of injury from a blast is complex and the risk imparted by an explosive device can vary across settings (e.g., enclosed versus open spaces) [22]. In principle, a blast wave in the form of a sphere of compressed and fast-expanding gases travels faster than sound from the center of the explosion and displaces and subsequently compresses an equal volume of surrounding air at high velocity [23]. In practice, however, blast effects can be more complex [23]. A blast wave can reflect from the ground or from an individual’s body with subsequent interactions between the primary and reflective waves that augment the initial wave, making it more complex and potentially more injurious. In complex environments and confined spaces, for example, the intensity of the blast wave can be magnified severalfold due to reflections from surrounding objects or walls, where persons in enclosed environments can suffer from more severe injuries than in open environments [23]. Thus, exposure to blast in complex environments can lead to polytrauma and a multisystem response.

In animal and post-mortem studies, structural changes have been found in diffuse brain regions following blast exposure, with preference for fluid-filled regions such as vascular structures, perivascular and periventricular regions, and the subpial glial plate [24]. Astroglial scarring where tissue interfaces with vasculature, cerebrospinal fluid, and at gray-white matter junctions has also been noted [25]. However, these abnormalities are not generally visible on typical clinical neuroimaging sequences, making them difficult to identify in real time. Another complicating factor in determining the neurologic effects of blast is that these often occur concomitant with other injuries. In addition to damage from the over pressurized wave itself, other factors related to the blast can also cause injury, such as objects being propelled by the blast, an individual being physically thrown into another object, and burns/inhalation of noxious chemicals [22]. Patients with blast injury frequently suffer damage to neurosensory organs (e.g., the ears and eyes) and to solid organs (e.g., heart and lungs), which can have direct and indirect influences on brain function [23]. Thus, determining the unique neurological impact of the blast wave itself is not always straightforward in clinical or research settings.

Given the inherent difficulties in studying RBE, novel biomarkers are needed to identify and better understand outcomes associated with blast exposure. Prior studies have identified transient changes in transcriptome profiles [26, 27] as well as blood-based neurotrauma biomarkers including neuronally-derived Tau, glial fibrillary acidic protein, ubiquitin carboxyl hydrolase (UCH)–L1, amyloid β (Aβ)–40, and Aβ-42 acutely following exposures to LLB during operational training [28–30]. Although DNA methylation studies in the same cohorts did not show alterations acutely following exposures to blast [31], altered DNA methylation was identified within genes involved in sleep and circadian rhythm chronically in individuals with a history cumulative blast exposures [30, 32, 33]. Specifically, work by Haghighi (author) and colleagues identified differentially methylated regions (DMRs) associated with high (≥40 exposures) versus low cumulative blast exposure [30, 32], with DMRs found in genes such as *NTSR1* (neurotensin receptor 1), associated with REM and anxiety-like behaviors in animal models [34]. Findings suggest a potential mechanistic link between cumulative RBE and dysregulated sleep.

DNA methylation is an attractive candidate mediator of enduring environmental effects on cellular function. Indeed, there is considerable evidence that environmentally regulated epigenetic states likely form the biological basis for gene × environment interactions. DNA methylation is a highly stable epigenetic mark that can be examined at genome-scale in clinical samples. Because blood samples are easily accessible and commonly available in epidemiological studies, many epigenome-wide association studies (EWAS) have been conducted in whole blood samples with the widespread application of Infinium Human Methylation Bead Chip assays, in line with the present study. Data from EWAS studies have been leveraged to identify a subset of CpG sites to create methylation risk scores (MRS), which can be used to predict the trait of interest in an independent cohort. MRS have been used as biomarkers for environmental exposures such as smoking [35, 36], and for prediction of individual risks of disease [37, 38] or treatment success [39], which has been widely adopted in cancer diagnosis and therapy [37–39]. Methylation risk scores have also been investigated in the context of poor mental health, including major depressive disorder (MDD) and PTSD which are frequent co-occurring conditions with blast and mTBI. Machine learning approaches have been used to train predictors of MDD status using DNA methylation data showing promising findings for MRS discrimination and prediction of MDD [40].

Growing evidence from animal models and human clinical studies suggests that changes to DNA methylation resulting from trauma are associated with PTSD [41–43]. A longitudinal EWAS study of PTSD reported DNA methylation alterations in CpG sites associated with immune related genes within the human leukocyte antigen region [44]. An EWAS study in Dutch military samples found that decreases in DNA methylation in various genomic regions across a period of exposure to combat trauma were related to increasing levels of PTSD symptoms, and these findings were replicated in an independent prospective cohort of US Marines [45]. Interestingly one of the regions with decreasing DNA methylation levels associated with PTSD symptoms included the *ZFP57* gene, which was shown to reverse methylation following successful PTSD treatment via trauma-focused psychotherapy [46]. This underscores the utility of DNA methylation not only as a potential risk marker of disease progression, but also as a putative marker of treatment response. Accumulating evidence linking epigenetic mechanisms with response to traumatic stress and the subsequent risk for PTSD [47–49] fits with our observed observations in Service Members with repeated exposures to blast and blast associated sequalae [26, 31]. Similarly, we aim to identify DNA methylation patterns associated with chronic exposures to blast in our Service Members in high-risk occupations, which we will then use to determine MRS predictive of blast exposures in our Veterans.

Building on results from prior work [26, 31], the purpose of this study is to carry out in-depth DNA methylation and functional gene expression experiments and phenotyping of sleep disturbance associated with cumulative blast exposures. This study will be completed in two phases. Data from Phase 1 were collected as part of a larger Walter Reed Army Institute of Research (WRAIR) protocol. We plan to expand upon- and refine the DNA methylation alterations previously identified by investigating additional cohorts of Military Service Members who are exposed to repeated blasts as part of their occupational duties (e.g., explosive breachers). We also aim to identify DNA methylation patterns that are associated with RBE as well as physiological and psychological symptoms in this population.

Because blast-related symptoms may develop and/or change over time, longitudinal studies comparing DNA methylations associated with evolving symptoms of cumulative blast exposure are needed. A study of this nature is not practical since recommendations are needed to address evolving symptoms for Veterans and Service Members with symptom presentation distal to the time of injury. Thus, in Phase 2, which is the focus of this manuscript, we will compare DNA methylation patterns found in Service Members with cumulative RBE with Veterans who experienced RBE as part of their occupational specialty.

We hypothesize that Veterans will exhibit similar DMRs associated with changes in sleep and other physiologic and psychological metrics following RBE. Veterans who were repeatedly exposed to blast as part of their occupational specialty will also exhibit altered DNA methylation patterns in the genes and genetic pathways identified in Service Members during Phase 1. Lastly, we will identify occupational specialties associated with higher risk for blast exposure among the Phase 2 cohort and compare occupational exposure between Phase 1 and 2 where available.

## Material and methods

### Study design

This is a cross-sectional observational study investigating DNA methylation patterns and transcriptional profiles associated with occupational exposure to blast. This study will be completed in two phases, with the first phase targeted towards active-duty Service Members. Data for Phase 1 have already been collected by our Walter Reed Army Institute of Research (WRAIR) collaborators and will be processed at the primary performance site, the James J. Peters VA Medical Center (JJP VAMC) in the Bronx, New York City. Phase 2 recruitment and data collection began on approximately 01/02/2023, and are currently ongoing. Phase 2 will be conducted among Veterans with exposure to repetitive blast during their lifetime. Participants will be recruited from the secondary performance site for this study, the VA Eastern Colorado Health Care System (ECHCS) in Colorado.

This study is being conducted according to the guidelines outlined in the Declaration of Helsinki. Study procedures involving human subjects for Phase 1 were approved by the Institutional Review Board (IRB) of WRAIR (Silver Spring, MD) and by JJPVAMC. All study procedures involving human participants for Phase 2 were jointly approved by the Colorado Multiple Institutional Review Board (COMIRB) and the JJPVAMC. Because methods being implemented for Phase 1 have already been described [26, 30, 31], we will focus here on Phase 2. However, Phase 1 methods are briefly described below.

### Summary of Phase 1 procedures

Data and blood samples for Phase 1 of the study were collected as part of a larger effort by WRAIR (protocols #2304 and #2838, Carr PI) in collaboration with Haghighi. Within these protocols, active-duty military Service Members were recruited as part of a multi-site study taking place across local and national military sites where, because of their occupation, participants routinely engaged in training courses in breaching and with a variety of weapon systems in varying complex environments involving repeated exposure to blast among personnel. Blood samples were collected at pre-, post-, and follow-up timepoints during the training courses for 265 breacher and 58 mortar weapons Service Members. Because the focus of this study was on cumulative exposure specifically, only data and samples for the pre-blast training timepoint will be used, which includes demographic data and basic medical information collected at study enrollment. See Table 2 for Inclusion and Exclusion Criteria.

**Table 2:** Inclusion and exclusion criteria for Phase 1.

**Inclusion criteria**
At least 18 years old
Active duty military personnel or civilian law enforcement personnel and actively involved in training or operations
Able to give informed consent
Subjects who are enrolled in the training course or assigned to participate in training exercises
Any individual who is fit for duty with no training restrictions
**Exclusion criteria**
Inability to read and sign consent
Not active duty military or civilian law enforcement personnel
Identified by command as being on any form of restricted duty
The PI (as per the IRB protocol) also maintains the prerogative to disqualify a volunteer if it is deemed that the volunteer’s participation would be unsafe for the volunteer or staff or would be disruptive to study conduct.

### Phase 2 procedures

#### Study population

Potential participants for Phase 2 will be up to 130 willing and eligible US military Veterans between the ages of 18 and 65 from the following populations: 1) those seeking outpatient mental health, rehabilitative, psychological, or other services within a VA Health Care System; 2) those in existing clinical and research databases; and 3) Veterans in the community not seeking care at the VA. This sample size was determined from a power analysis see below under the “Sample size” section. Inclusion and exclusion criteria are presented in Table 3.

**Table 3:** Inclusion and exclusion criteria for Phase 2.

**Inclusion criteria**
Age between 18 and 65
Veterans with MOSs during service defined to be high-risk that are associated with exposures to blast during training and/or combat. These can include Cannon Crewmembers, Explosive Ordinance Disposal Specialists, Fire Infantrymen, Combat Engineers, and Special Forces etc.
Exposure to repeated blasts as part of military duties
Score of 2 of greater on at least one Neurobehavioral Symptom Questionnaire (NSI) symptom
Ability to provide informed consent
Willingness to provide blood
**Exclusion criteria**
Inability to adequately respond to questions regarding the informed consent procedure
Currently involved in the criminal justice system as a prisoner or ward of the state
Non-English speaking
Current occupation involving shift work
Female Veteran who is pregnant
Lifetime history of bipolar disorder or psychosis
History of moderate or severe TBI
Changes in regularly used medications (e.g., daily) in the last month for medications known to impact sleep (e.g., benzodiazepines, etc.)
Current chronic steroid use
History of neurological or neurodegenerative disease such as multiple sclerosis
History of brain tumor
History of seizure disorder unrelated to TBI
History of HIV/AIDS
Current systemic illness such as uncontrolled diabetes mellitus, hypo- or hyperthyroidism, or systemic cancer
Acute disease at the time of enrollment (defer sampling until subject recovers). Acute disease is defined as the presence of a moderate or severe illness with or without fever

#### Recruitment and screening

The research team will work with providers within the ECHCS to enlist their assistance with recruitment. Potential participants will be informed about the research project via letter, flyer, and/or presentation. Professionals treating patients will be asked to aid in this process. Research team members will attend team meetings and brief staff about the study, as well as send potential participants direct mailings regarding the study. In addition to recruiting from facilities within the ECHCS, the research team will recruit Veterans in the community who are eligible to receive Veterans Health Administration (VHA) care.

Individuals who express interest in participating in the study will participate in a brief screening process. A member of the research team will conduct the screening process via telephone or in-person. Screening will also include a review of VA electronic medical records. Those who are eligible and willing to participate will be scheduled to complete the informed consent and study session. Written informed consent will be obtained by trained study team members from all participants at the beginning of their study session. All members of the research team will have been trained in COMIRB procedures and will be under the direct supervision of Dr. Brenner. The nature of the study and potential risks and benefits will be discussed, and participants will have the opportunity to ask questions.

#### Assessment and outcome measures

We will perform a comprehensive clinical assessment battery, which is outlined in Table 4 below. We will also collect up to 35ml of blood from each participant to investigate genome-scale DNA methylation and gene transcript profiles.

**Table 4:** Study measures Phase 2.

Measure	Time (min)	Domain
University of Washington Suicide Risk/Distress Assessment (UWRAP)	10	Safety
Demographics and Blast Exposure-Related History	5	Diagnostic
Structured Clinical Interview for DSM-5 (SCID-5)	90	Diagnostic
Structured Clinical Interview for Sleep Disorders Revised (SCISD-R)	30	Diagnostic
Clinician-Administered PTSD Scale for DSM-5 (CAPS-5)	30	Diagnostic
Boston Assessment of Traumatic Brain Injury Lifetime (BAT-L)	15	Diagnostic
Columbia-Suicide Severity Rating Scale (CSSRS)	10	Suicide
Blast Ordnance and Occupational Exposure Measure (BOOM)	8	Blast Exposure
Insomnia Severity Index (ISI)	3	Sleep
Pittsburgh Sleep Quality Index (PSQI)	5	Sleep
Epworth Sleepiness Scale (ESS)	2	Sleep
Smith Morningness-Eveningness Scale (M-E)	5	Sleep
Fatigue (PROMIS)	2	Fatigue
Trauma-Related Nightmare Survey (TRNS)	5	Nightmares
Combat Exposure Scale (CES)	5	Stress
Beck Depression Inventory (BDI)	3	Mood
Beck Anxiety Inventory (BAI)	3	Mood
PTSD Checklist for DSM-V (PCL-5)	3	PTSD
Paced Auditory Serial Addition Test (PASAT)	15	Neurocognitive Performance
Gradual Onset Continuous Performance Task (gradCPT)	8	Neurocognitive Performance
Test of Malingering Memory (TOMM)	20	Neurocognitive Performance
Neurobehavioral Symptom Inventory (NSI)	5	Post-Concussive Symptoms
Headache Impact Test (HIT-6)	5	Headaches
Pain, Enjoyment of Life, and General Activity Scale (PEG)	1	Pain
Tinnitus Handicap Inventory (THI)	5	Tinnitus

#### Screening for repetitive blast exposure

For Phase 2 of the study, we created a modified version of the Blast Ordnance and Occupational Exposure Measure (BOOM) for Self-Reported Lifetime Blast Exposures for our screening process. Because we are primarily interested in repeated exposure to blast, the modified BOOM questions assess for the approximate lifetime blast exposures, with the selection options being “None; 1; and 2 or more.” We then ask whether the participant was able to physically feel the blast wave, with the answer options being “No; Yes, but only once; and Yes, greater than one time.” If the potential participant had more than one exposure in aggregate across categories, and recalled feeling the blast more than one time, they would be considered eligible based on the criteria of having had repeated blast exposure in their lifetime. See S1 Table for the Modified BOOM. In addition to the modified version of the BOOM used for screening, we have included the full BOOM as a measure should the participant be considered eligible for the study.

## Biomarker experiments and data analyses

We will perform cross-comparison of DNA methylation and transcriptional alterations associated with cumulative blast in Service Members and Veterans. Specifically, DNA methylation and associated gene expression alterations identified from Phase 1 will be investigated in our Phase 2 cohort, which includes Veterans with a history of RBE as part of their military occupation (see Table 1). We will examine DNA methylation and gene transcript profiles using the same experimental platforms described in Phase 1 [26, 31]. The sample and data processing for the DNA methylation and RNA-sequencing data, as well as QC procedures and bioinformatics approaches, will also be analogous to those described in Phase 1 [26, 31], and will be briefly described below. For Phase 2, blast exposure will be used as a quantitative measure in statistical models.

### Sample size

In Phase 2, the Veteran high-risk occupation sample to be recruited will be n = 130, based on the following power calculations for the primary biomarker to be investigated i.e., DNA methylation, using an approximation algorithm provided in the R library “ssize.fdr”. The primary hypothesis tests a quantitative predictor while the power calculator is designed to test group differences, thus we approximated the power with a 6-group loop design, meaning comparing 6 groups of incrementally increased exposure severity. In previous work [26, 31], we estimated the 90th percentile of the empirical genome-wide standard deviation distribution of the beta value to be σ = 0.1. We allowed 12 degrees of freedom for the covarying of 10 principal components to adjust for population stratification and age and sex With these parameters, we will have >80% power to detect effect sizes of Δ = 0.075 or larger, or d/sigma = 0.75, a moderate to large effect size, for a total sample size of N = 130.

### DNA methylation sample processing and quality control

Isolated genomic DNA will be bisulfite converted (Zymo Research) and CpG dinucleotide methylation will be determined using Illumina Infinium Human Methylation EPIC BeadChip microarrays (described previously [33]). Data and QC analyses will be performed using the open-software program R, v4.3.1 [534, an environment for statistical computing, and Bioconductor 2.17 [50], and all raw data files (.idat) will be processed by the minfi package [34]. All samples will undergo quality control processes for sample tracking and sex prediction analyses previously described [26, 31].

### DNA methylation data analysis

We will use the matrix of M-values (logit transformation of beta-values), which correspond to methylation levels. Surrogate variable analysis (SVA) will be performed to add surrogate variables to rule out potential batch effects. For the DMRs identified in Service Members (as per approach previously described [26, 31]), we will determine whether DNA methylation levels within these DMRs correlate in Veterans with relative cumulative blast exposures in a dose-response fashion. Specifically, we will fit spline models of increasing complexity to the M value by the amount of blast exposure for each subject, adjusted for covariates i.e., age and history of TBI, cell heterogeneity, and population stratification variables in the model. We will compare successive models using analysis of variance to select the most parsimonious well-fitting model. It should be noted that due to the cellular heterogeneity of whole blood as a sample, we considered that cell proportion may be a potential confounder between methylation and our factors of interest. Therefore, we will use the estimateCellCounts function in the R package minfi [51, 52], with the most updated reference data (i.e., FlowSorted.Blood.EPIC), corresponding to the EPIC BeadChip platform, to calculate the cell proportion estimates of six cell types (CD4 T-cell, CD8 T-cell, natural killer, B-cell, monocytes and granulocytes), with estimates being highly correlated across both generations of the Illumina HumanMethylation 450K and EPIC BeadChips [53]. We will compare each cell type’s proportions between groups in our analyses (as relevant for all aims here). If the estimated cell proportions differ between these group comparisons, then the cell estimates will be included in the analytic models as covariates to adjust for potential confounds. We will adjust for population stratification in the methylation data by performing a principal component analysis on the methylation values from pruned CpGs located close to SNPs, as described in Barfield et al., and we will include the top 2 factors as covariates in all analyses [54].

### RNA sample/library preparation and sequencing for gene transcript profiling

Following total RNA isolation, globin mRNA will be removed with Globin Clear Human Globin mRNA Removal kit (Ambion, Inc.). Total RNA sequencing libraries will be prepared from RNA samples with RNA integrity numbers (RIN) ≥6.0 using the Illumina Stranded Total RNA Library Prep Kit with Ribo-Zero Gold in accordance with the manufacturer’s instructions. Briefly, 290ng-500ng of total RNA will be used for ribosomal depletion and fragmented by divalent cations under elevated temperatures. The fragmented RNA undergoes first-strand synthesis using reverse transcriptase and random primers followed by second-strand synthesis to generate cDNA. The cDNA fragments will undergo end repair, adenylation, and ligation of Illumina sequencing adapters. The cDNA library will be enriched using 11 cycles of PCR and purified. Final libraries will then be evaluated using PicoGreen (Life Technologies) and Fragment Analyzer (Advanced Analytics) and will be sequenced on an Illumina HiSeq2500 sequencer (v4 chemistry) using 2 x 125bp read lengths.

### RNA-seq data preprocessing and bioinformatics analysis gene transcript profiling

RNA sequencing reads will be aligned to the human reference genome using STAR aligner (v2.7.5b) [55]. Quantification of genes annotated in GRCh38.p13 will be performed using feature Counts (v2.0.1). QC metrics will then be collected with Picard (v2.22.3) and RSeQC [56] followed by normalization of feature counts. We will use the voom function in limma to get logCPM matrix, where the design matrix consists of intercept, age, history of TBI, and variable of interest, i.e., blast exposure dosage. We will use SVA to rule out potential batch effects. Analyses will be performed in limma. Point-wise and multiple testing adjusted p-values will be reported. Differentially expressed genes identified in Service Members that associate with cumulative blast exposure, will be used to perform a dose-response analysis by exposure severity, like that described for the methylation analysis.

### Assessing RBE associated methylation and transcriptional profiles with clinical symptoms

In exploratory analyses, we will conduct regression analyses to determine whether RBE DNA methylation and Transcriptional profiles as above are associated with a spectrum of symptom severity in our Veterans, specifically, symptoms of PTSD, depression, pain, neurocognitive functioning, and sleep disturbance severity, adjusting for covariates (i.e., age, sex, history of TBI, cell heterogeneity, and population stratification variables in the model). Association between these symptoms and the cumulative exposure will be tested in dose-response models, as described above. Given the high incidence of sleep disturbance in Veterans with blast exposure, we will focus on clinical sleep outcomes related to common behavioral sleep disturbances and their impact on daytime function, degree of insomnia symptomology, circadian preferences, sleep quality, nightmares, fatigue, and daytime sleepiness. These will be tested for association with cumulative RBE.

#### Testing DNA methylation risk scores

In the active duty sample (Phase I), CpG sites with significant association with blast exposure will be used in conjunction with machine learning algorithms to create methylation risk scores (MRS). MRS have been used as biomarkers for environmental exposures such as smoking [35, 36], and for prediction of individual risks of disease [37, 38] or treatment success [39]. Methylation risk scores have also been investigated in the context of poor mental health, including MDD and PTSD which are frequent co-occurring conditions with blast and mTBI. Machine learning approaches have been used to train predictors of MDD status using DNA methylation data showing promising findings for MRS discrimination and prediction of MDD [46], which will also be adopted in the present study. The rule base developed for the MRS RBE prediction, derived in the Active Duty sample (Phase I), will be transferred and tested in the Veteran sample with respect to RBE prediction (Phase II).

## Discussion

In this study we will investigate repeated exposures to blast in a real-world operational setting that will lead to discovery of novel stable DNA methylation markers of cumulative blast in Service Members (Phase 1) and Veterans (Phase 2). During Phase 1 (now completed), we collected a critical mass of difficult-to-obtain samples from a large cohort of Service Members with RBEs, allowing us to identify DNA methylation signatures of blast that track with associated chronic symptoms in Service Members. In Phase 2, which is currently in progress, we will collect similar samples in Veterans that will allow us to analyze these same DNA methylation signatures in this cohort. The aim is to identify biomarkers (i.e., DNA methylation signatures that correspond to transcriptional changes that associate with chronic exposures to blast, as well as RBE methylation risk scores in our Service Members with known high-risk occupation that can then be used as biomarkers predictive of blast exposure effects in our Veterans.

The expected therapeutic and rehabilitative impact of these findings is significant since these blast-associated DNA methylation loci and MRSs can be used in future clinical trials as objective markers of treatment response. Furthermore, as with PTSD [46], treatment of chronic blast-related symptoms, such as sleep disturbance, may be reflected in biological regulation and, as such, is likely to be accompanied by specific epigenetic changes [57, 58], underscoring the utility of DNA methylation not only as a potential risk marker of disease progression but also as a putative marker of treatment response. As such, we will also include other potential factors that may impact methylation markers in our analyses, such as mental health symptoms (e.g., symptoms of PTSD, depression, anxiety), combat exposure, sleep disorders, headaches, pain, tinnitus, history of lifetime mild traumatic brain injury, and neurobehavioral symptoms.

Additionally, we will identify specific occupational specialties across branches of the military with a higher incidence of blast exposure among our Veteran participants. Given the limited work to date on risk profiles within a given occupational specialty across military branches, this study will be an important addition to the literature in terms of better understanding different types of blast exposures across occupational specialties. We will also compare outcomes among similar occupational specialties between Service Members and Veterans where available (e.g., explosive breachers). This is expected to increase understanding regarding the manner in which combinations of different types of blast exposures, including low level blast, high level blast, and exposure to various weapons across both in training and combat settings are associated with long-term negative outcomes.

The potential benefits to the military and Veteran population from this study are considerable. Blast-related head injuries are a major problem in VA and military populations and have attracted substantial attention at the national level. Understanding how biological and molecular changes associated with chronic blast exposure will help not only to identify those at risk but will also be important in determining treatment options to mitigate deleterious long-term sequelae. Specifically, the proposed project aims to identify DNA methylation risk markers to distinguish those at risk before debilitating symptoms related to repeated blast exposure can potentially emerge; thereby allowing for preventative intervention. These same markers can also be used to target Veterans with histories of repeated blast exposure in future clinical trials to measure treatment outcomes of co-occurring blast-related symptoms.

### Limitations

The proposed study has limitations. We will be unable to account for an individual military personnel’s genetic risk factors and predisposition to responsivity to blast exposure, as blast exposures are to be examined via transcriptional regulatory profiles. Our WRAIR collaborators are in the process of genotyping a panel of genetic loci associated with TBI and blast that can also be incorporated in our studies to determine whether the transcriptional regulatory changes observed in these cohorts may be driven by genetic risk variance. Additionally, focused investigations of transcriptional regulatory signatures of blast are not representative of other potentially important biomarkers such as proteins, metabolites, miRNA etc. that may contribute to molecular pathophysiology of cumulative blast exposures and related sequalae. Phase 1 lacks representation of both sexes, as almost all participants engaged in the breacher- and mortar training courses identified as male. As such, the data from Phase 1 may not be generalizable to the relatively small (but growing) population of female Service Members and Veterans. The increase in female population in the US Armed Forces and recent inclusion of women in combat roles warrant investigation of sex differences and can be the focus of future studies. Additionally, we will be targeting recruitment for both female and male Veterans in Phase 2. Lastly, Phase 2 data were collected in a single geographical region, which may also limit the generalizability and somewhat limit the type of military occupations accounted for by those who currently reside in the state of Colorado.

## Conclusions

In summary, the study outlined above is expected to provide data on a novel marker of repetitive blast exposure by identifying DNA methylation patterns. We will analyze the relationship between these methylation patterns and chronic effects of blast exposure, including sleep disruption, and other psychological and physiological symptoms. We will also be able to associate these changes with different MOSs to better understand occupations associated with a higher risk of blast exposure. This work will help to inform the growing body of research on long-term effects of occupational blast exposure as well as areas such as combat readiness, protocols for limiting blast exposure in training and combat settings, as well as prevention and treatment to improve long-term outcomes in Service Members and Veterans.

## Supporting information

S1 Table Modified BOOM–Blast exposure. (DOCX)
