# Supplementary material for: Study protocol: Identifying transcriptional regulatory alterations of chronic effects of blast and disturbed sleep in United States Veterans
Source: PLoS One. 2024 Mar 27;19(3):e0301026. doi: 10.1371/journal.pone.0301026 (PMC10971577; doi:10.1371/journal.pone.0301026)
Supplement: S1 Table — (DOCX) [file pone.0301026.s001.docx]

**S1 Table. Modified BOOM – Blast Exposure**

| **INSTRUCTIONS**: Please use the table below to help us quantify your approximate lifetime blast exposure. For the purpose of this survey, “exposure” is defined as any incident where you physically felt the blast/overpressure wave whether the blast was the result of an incoming enemy fire or a charge or weapon system that you fired or were near to. Exposure does not mean events in which you observed a blast but did not feel its pressure wave. Please note that we are only looking for gross estimates. | | | | |
| --- | --- | --- | --- | --- |
| **For the following ordnances:** | | **Select the approximate range of lifetime blast exposures (see definition above)** | **Did you physically feel the blast?** | **Notes** |
| Small explosives | ***CIRCLE ANY APPLICABLE*** | None  1  2 or more | No  Yes, but only 1x  Yes, >1x |  |
|  | *e.g., grenades, flash bangs, landmines, claymores* |  |  |  |
| Shoulder-fired weapons | ***CIRCLE ANY APPLICABLE*** | None  1  2 or more | No  Yes, but only 1x  Yes, >1x |  |
|  | *e.g., RPG, LAW, SMAW, Dragon, GUSTAV, AT4, Javelin, grenade launcher* |  |  |  |
| Explosive breaching |  | None  1  2 or more | No  Yes, but only 1x  Yes, >1x |  |
| Explosive ordnance disposal or blast-related demolition |  | None  1  2 or more | No  Yes, but only 1x  Yes, >1x |  |
| Improvised Explosive Devices (IEDs) |  | None  1  2 or more | No  Yes, but only 1x  Yes, >1x |  |
